# Optimizing Emergency Department Length of Stay and Quality of Care: A Quality Improvement Project

**DOI:** 10.7759/cureus.71989

**Published:** 2024-10-21

**Authors:** Mohammad H Al-Na'seh, Ahmad M Elheet, Ali M Alhayek, Albatool T Sabri, Ahmad K Al Owaidat

**Affiliations:** 1 School of Medicine, The University of Jordan, Amman, JOR; 2 Department of Emergency Medicine, Jordan University Hospital, Amman, JOR

**Keywords:** audit, consultation time, ed crowding, emergency department disposition, hospital length of stay (los)

## Abstract

Background

Patients’ long length of stay (LOS) in the emergency department (ED) is a common measure of quality of care that could be a cause of increased morbidity and mortality. This quality improvement project (QIP) aimed to identify causes of long LOS and to improve common causes such as long consultation times.

Methodology

Over two months, three plan, do, study, and act (PDSA) cycles were conducted aiming to identify causes of long ED LOS and improve common causes that extended ED LOS beyond four hours. Additionally, the project aimed to reduce the time taken from requesting a consultation from another specialty until it was completed and documented to an average of 60 minutes. Main interventions included raising awareness of staff and administration through video presentations, printed posters, direct contact, and optimizing the electronic health records system to audit performance.

Results

From PDSA cycle 1 through PDSA cycle 3, average consultation times decreased from 91 to 65 minutes. Common organization-related causes of long LOS included pending radiology or laboratory investigations, awaiting inpatient admission, and awaiting consultations from other specialties. Physician-related factors included delay in documentation likely resulting from heavy workload. Pending investigations and admissions were factors that could be amended with better administrative control.

Conclusions

A multifaceted approach that tackles physician-related and organization-related factors could be a necessity to improve LOS and the quality of care in the ED. Having staff and administration aware of targets and performance along with utilizing the electronic health records system to audit performance and increase efficiency are beneficial in improving the LOS in the ED.

## Introduction

Emergency department (ED) length of stay (LOS) is the time interval from a patient’s arrival until departure from the ED. It is a measure of performance and quality of care in hospitals [1]. Long LOS and ED overcrowding are often multifactorial, with common causes including patients’ old age, inadequate staffing, delay in admission, delay in consultations, and delay in laboratory or radiological investigations [2-5].

Long ED LOS and ED overcrowding lead to adverse health outcomes and increased mortality due to delays in treatment and poor quality of care, nonadherence to guidelines, patient and staff dissatisfaction, and increased healthcare spending [5-9]. Proposed solutions to tackle long LOS included extending primary care hours and establishing timed patient disposition targets [5]; such patient disposition targets included a target ED LOS of less than four hours in the United Kingdom and Australia [10,11] and six hours in New Zealand [12]. This quality improvement project (QIP) aimed to discover factors that prolonged patients’ ED LOS and to implement changes via the Plan, Do, Study, and Act (PDSA) methodology to reduce LOS and improve the quality of care provided in the ED at Jordan University Hospital (JUH), a tertiary teaching hospital.

This project was previously presented as a poster at the Jordanian Doctors in the United Kingdom (JDUK) Second International Conference on August 25th to 26th, 2024.

## Materials and methods

Study setting

We undertook this QIP in the ED at JUH over 72 days in 2022 as a team of three final-year medical students, the chief emergency medicine resident doctor, and an emergency medicine specialist doctor. The ED at JUH has around 46 beds, with usually around five resident doctors and one consultant working on the shift. Around 100,000 patients visit the ED annually. The QIP was conducted in cooperation with the quality assurance (QA) department and the information technology (IT) department.

Measures

The primary objectives of this 72-day-long QIP were to identify causes of long ED LOS and improve common causes that extended ED LOS beyond four hours. Additionally, due to previously reported findings that long consultation times cause extended LOS [2,4], we decided to approach this factor as a separate objective from the first cycle. The project aimed to reduce the time taken from requesting a consultation from another specialty until it was completed and documented (consultation time) to an average of 60 minutes.

Secondary objectives to improve the quality of care in the ED apart from reducing ED LOS and consultations times were to ensure legible data documentation and standardization of medical practice. For this, the plan was to implement a system that would ensure adherence to clinical guidelines for specific diseases and encourage more legible data documentation.

To accomplish our goals, it was necessary to gather reasons for delay beyond the four-hour target. Moreover, we needed to measure the time taken from a consultation’s request until it was completed and documented. We also had to monitor the data documented for ED patients and the use of the guidelines set on the electronic health records (EHR) system.

Strategy

To improve LOS and quality of care in the ED, specifically in terms of identifying causes of long LOS, cutting down consultation times, encouraging legible data documentation, and promoting standardized, evidence-based practice, we aimed for a multifaceted approach that addresses physician-related and organization-related factors. We made physicians aware of the targets, encouraged them to achieve the targets, and informed those who did not. This project also focused on utilizing the EHR system to facilitate detecting causes of long LOS, monitoring consultation times, and providing readily accessible guidelines to treat common emergencies with standardized high-quality care.

**Figure 1:**
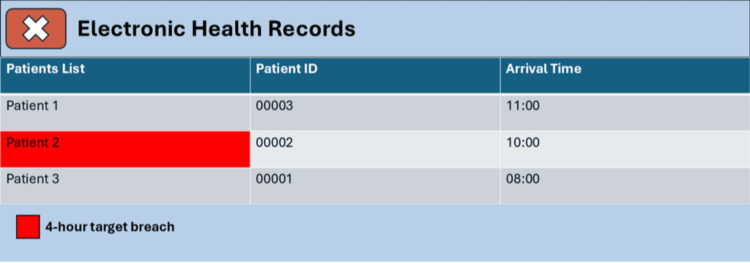
Example of the target breach alert.

In cooperation with the IT department, we made a few modifications to the EHR system in the first cycle. For instance, to a red alert was made to show in the current patient list to notify that the patient’s LOS exceeded four hours (Figure 1).

Furthermore, we made it so that it would be required to document reasons for delay before being able to open the patient’s notes, request tests, view results, or document any further. Further, a timer would appear inside each patient’s notes to indicate how much time remained until the four-hour target was breached.

The consultations were requested and documented through a specific area in patients’ notes. We modified it so it would record the timing of the process so that it could be monitored.

For the secondary objectives, doctors at JUH usually improperly documented everything in one section of the notes, which hindered finding critical information promptly when needed. Hence, we ensured that the notes could be segmented into fields that would require filling so that the information would be more legible. Required fields were Chief Complaint, History of Presenting Illness (HPI), Physical Exam, and Plan.

Moreover, a section that would activate a protocol of treatment for specific diseases was inserted into each patient’s notes so that doctors would be able to use them as guidance to standardize treatment, providing high-quality care. We inserted a “Protocols” button that would open a window with steps to treat specific conditions such as diabetic ketoacidosis, sepsis, myocardial infarction, and major trauma. The steps were listed as a checklist with room for notes and a time recorder.

We then made a video presentation to all physicians working in the ED explaining the changes and that their compliance would be monitored to fulfill our objectives.

The process of planning and setting up the project started on the first of March 2022, and the first auditing process happened between March 15 and April 1, 2022.

In the second cycle, we re-sent the video presentation to all JUH doctors and designed a poster indicative of the audit objectives. The posters aimed to remind physicians of the ongoing audit and emphasized some of the objectives being monitored such as encouraging using the new Protocols sections and documenting information in the designated fields. The posters were posted in each bay of the ED. The second cycle spanned the period between April 1 and April 28, 2022.

For the third cycle, which occurred between April 28 and May 12, 2022, in addition to repeating the previous interventions, the department heads were contacted by the QA department so that the departments with lower compliance rates were aware and encouraged to improve.

Ethical considerations

This project did not meet the criteria to be classified as research and thus did not require any ethical approval from the research committee as patients were not involved in the planning or design of this QIP. However, it was locally registered as a QIP with the QA department as one of the “FOCUS PDSA” projects.

## Results

**Figure 2:**
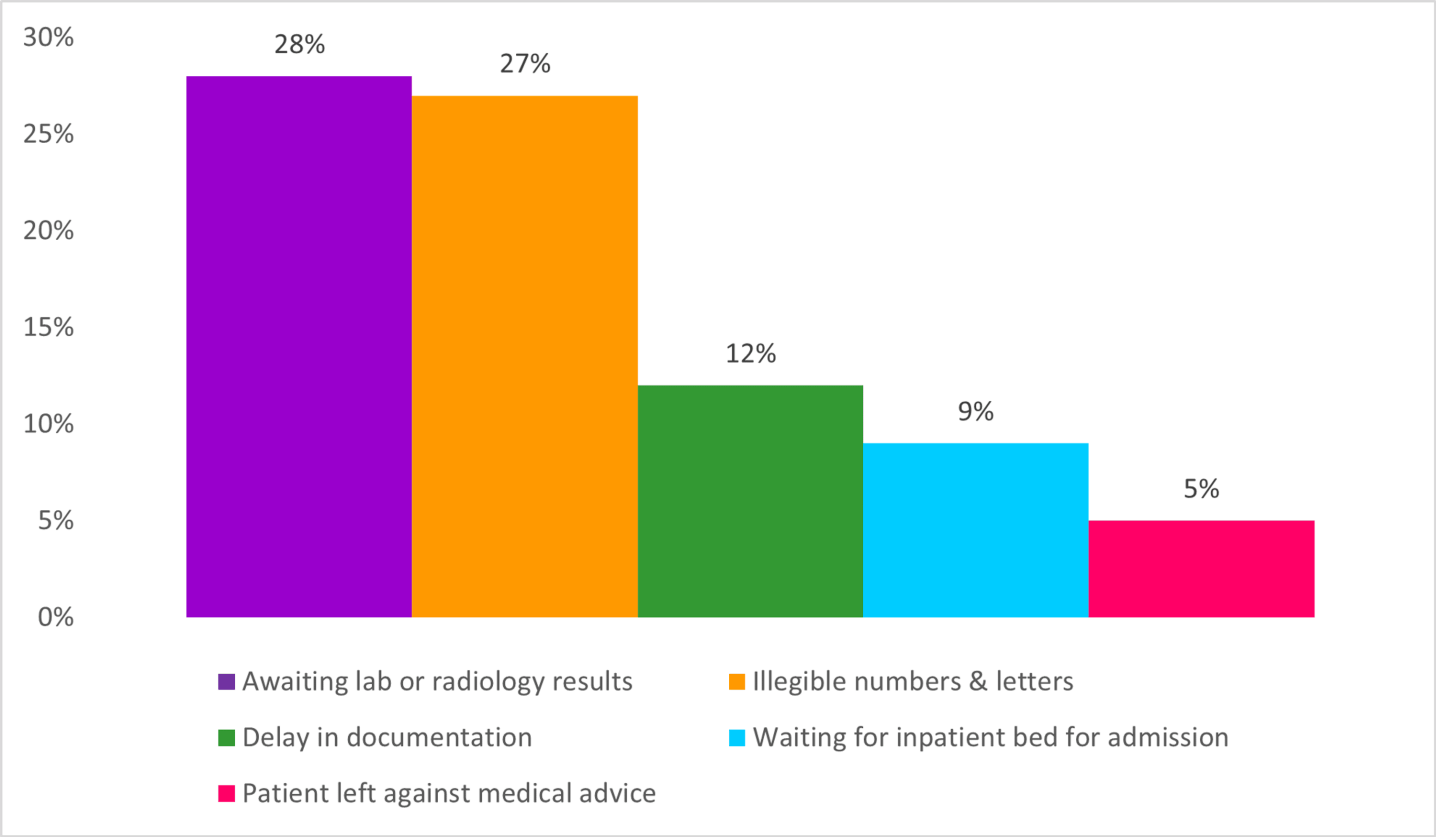
Documented reasons for delay in the first cycle. The data have been represented as percentages out of a total number of 301 long length of stay cases.

For the first cycle, there were 301 cases of long LOS. The most common entries for delay reasons can be seen in Figure 2. Moreover, there were 486 consultations in total for patients in the ED, with the longest consultation taking 41.7 hours to complete. In the process of auditing legible data documentation, 58.6% out of 150 random notes from this cycle had missing data or unfilled sections, with random letters or numbers being inserted in required fields. Unfortunately, there was no way to track and audit the use of the protocols system. We also observed a habit of entering random, illegible numbers or letters as reasons for breaching the four-hour target to bypass documenting delay reasons and be able to proceed with patient management instead.

The specialties that had the longest consultation times were General Surgery (average 202 minutes), Internal Medicine (average 126 minutes), Urology (average 92 minutes), Pediatrics (average 84 minutes), and Obstetrics & Gynecology (average 57 minutes).

**Figure 3:**
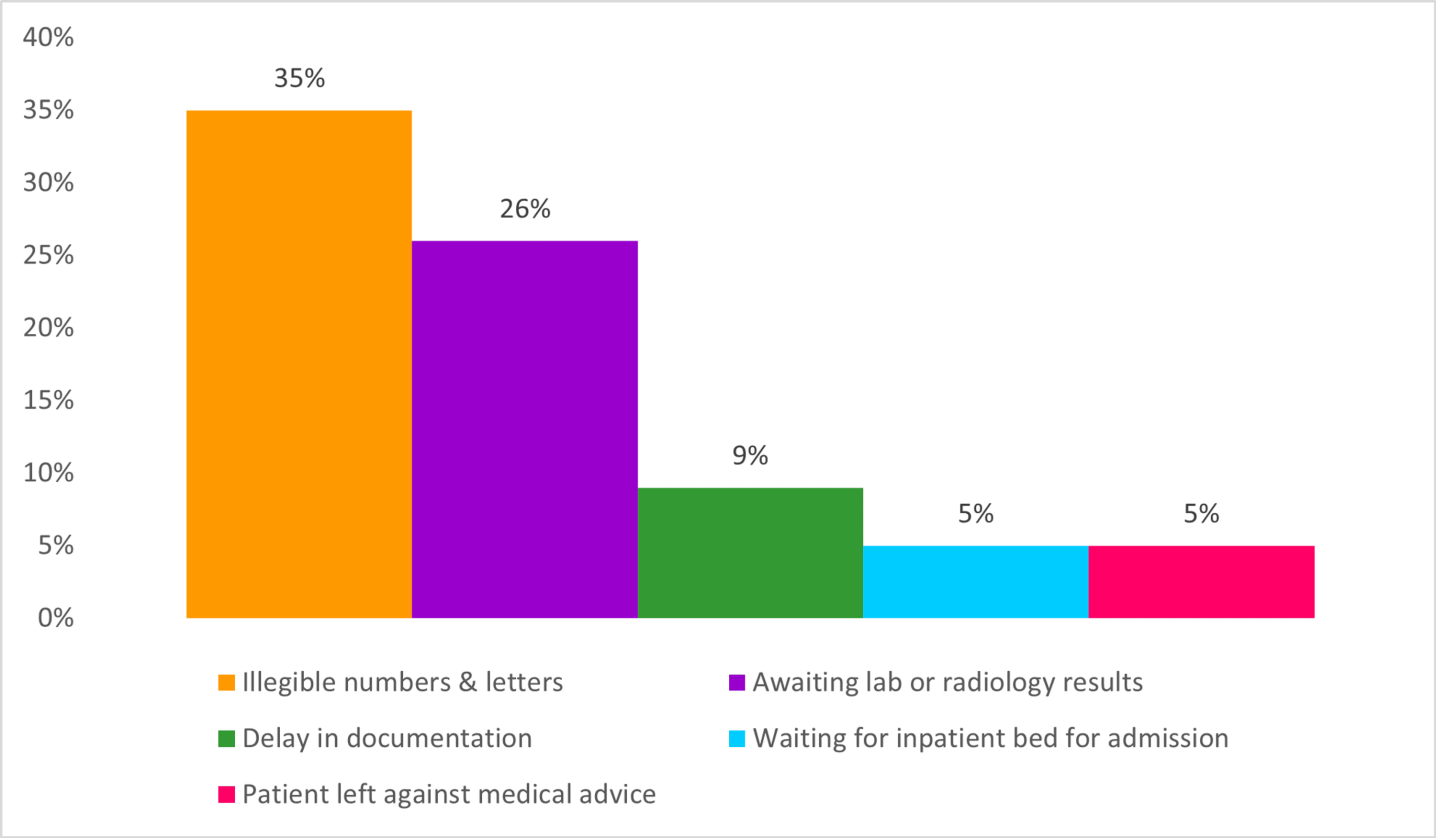
Documented reasons for delay in the second cycle. The data have been represented as percentages out of a total number of 335 long length of stay cases.

In the second cycle, there were 335 cases of long LOS. The most common entries for delay reasons can be seen in Figure 3. In addition, a total of 745 consultations were conducted for patients in the ED, with the longest consultation taking 57.48 hours to complete. It is important to note that one consultation had taken significantly longer to complete, which affected the list of the specialties with the longest consultation times. Moreover, while monitoring legible data documentation, 62.5% out of 150 random notes from this cycle had missing data or unfilled sections, with random letters or numbers being inserted in required fields.

In this cycle, the specialties that had the longest consultation times were Internal Medicine (average 119 minutes), General Surgery (average 112 minutes), Urology (average 108 minutes), Orthopedics (average 62 minutes, 29 minutes excluding one outlier), ENT (average 34 minutes), Obstetrics & Gynecology (average 29 minutes), and Pediatrics (average 15 minutes).

**Figure 4:**
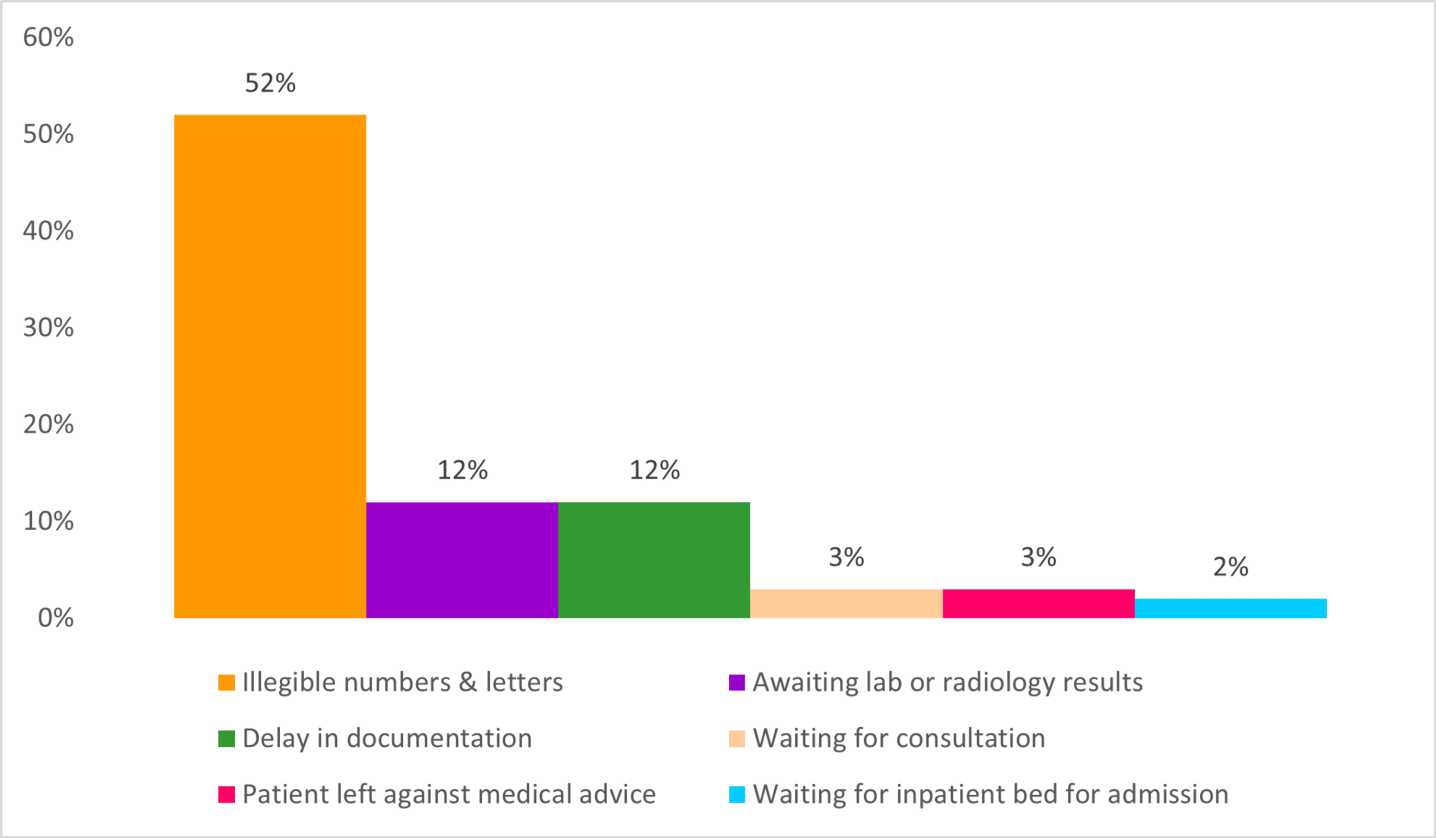
Documented reasons for delay in the third cycle. The data have been represented as percentages out of a total number of 263 long length of stay cases.

For the third cycle, there were 263 cases of long LOS. The most common entries for delay reasons can be seen in Figure 4. Moreover, a total of 428 consultations were conducted for patients in the ED, with the longest consultation taking 23.94 hours to complete. Additionally, while monitoring legible data documentation, 55.5% out of 150 random notes from this cycle had missing data or unfilled sections, with random letters or numbers being inserted in required fields.

In this cycle, the specialties that had the longest consultation times were ENT (average 100 minutes), General Surgery (average 98 minutes, 58 minutes excluding two outliers), Urology (average 91 minutes), Internal Medicine (average 80 minutes), Obstetrics & Gynecology (average 65 minutes, 34 minutes excluding one outlier), and Pediatrics (average 32 minutes).

Table 1 shows the difference between the three cycles in terms of mean and maximum consultation times.

**Table 1:** Comparison of consultation time between cycles. *: Mean and maximum consultation times in the second cycle were affected by the presence of one outlier case; the parentheses indicate the values after excluding the outlier.

	Cycle 1	Cycle 2	Cycle 3
Mean consultation time (minutes)	91	75* (70)	65
Maximum consultation time (hours)	41.7	57.48* (29.38)	23.94

## Discussion

An improvement in consultation times from an average of 91 minutes to 65 minutes was achieved following raising awareness of targets, auditing through the EHR system, and informing heads of departments of the performance. Organizational factors such as waiting for admission or radiology/laboratory results were improved following reporting to administration and related departments, who, on their part, ensured that all relevant staff members were aware of the issue and their collective performance and encouraged to enhance it. Unfortunately, there was no way to monitor the use of the added protocols because there was no way to detect all cases of, for example, diabetic ketoacidosis and to check the use of the protocol. Although the gross number of long LOS cases was lower with each PDSA cycle, the cycles varied in length, and the average number of patients attending the ED differed every month.

Common causes of long LOS, as reported by physicians, included awaiting radiology/laboratory results, awaiting inpatient admission, and awaiting consultation from other specialties, which is in line with findings of previous research [2-5]. It could be inferred from the data that physicians were working under a heavy workload, which led them to bypass documenting delay reasons to be able to go on with patient management instead of taking their time to document the reasons for the delay.

A commonly reported cause for long LOS was a delay in documentation. A possible explanation could be a heavy workload leading physicians to attend to patients with more urgent needs instead of documenting and discharging stable patients as documenting on EHR is a reported cause of increased workload and need to work overtime [13] and, as can be seen in this QIP, seemed to be an inconvenience that led physicians to document illegible letters or numbers instead of documenting actual reasons for delay in the ED. Furthermore, the consistent finding of physicians documenting all information in one section instead of taking the time to organize and segment the data into separate fields could also indicate the inconvenience caused by EHR documentation.

Limitations of this project included not being able to audit the use of the added protocols, along with the fact that this project was conducted in one center, meaning that our findings cannot be generalized to other centers. Moreover, due to confounding circumstances such as case severity that could play a role in LOS, it is sometimes necessary to analyze individual cases on their own, as indicated by the presence of outlier cases in consultation times.

Routine auditing and keeping staff aware of performance to ensure consistent improvement is recommended. Furthermore, we recommend taking into consideration organization-related, staff-related, and patient-related factors when trying to tackle the issue of LOS in the future. This QIP was followed on with further projects aiming to re-audit for our objectives and to increase the number of conditions in the protocols section.

## Conclusions

This project demonstrated that a multifaceted approach that tackled physician-related and organization-related factors could be necessary to improve LOS and the quality of care in the ED. Common organization-related causes of long LOS included pending radiology or laboratory investigations, awaiting inpatient admission, and awaiting consultations. Physician-related factors included delay in documentation likely resulting from heavy workload. Having staff and administration aware of targets and performance along with utilizing the electronic health records system to audit performance and increase efficiency are beneficial in improving the LOS in the ED.

## Figures and Tables

**Figure 5:**
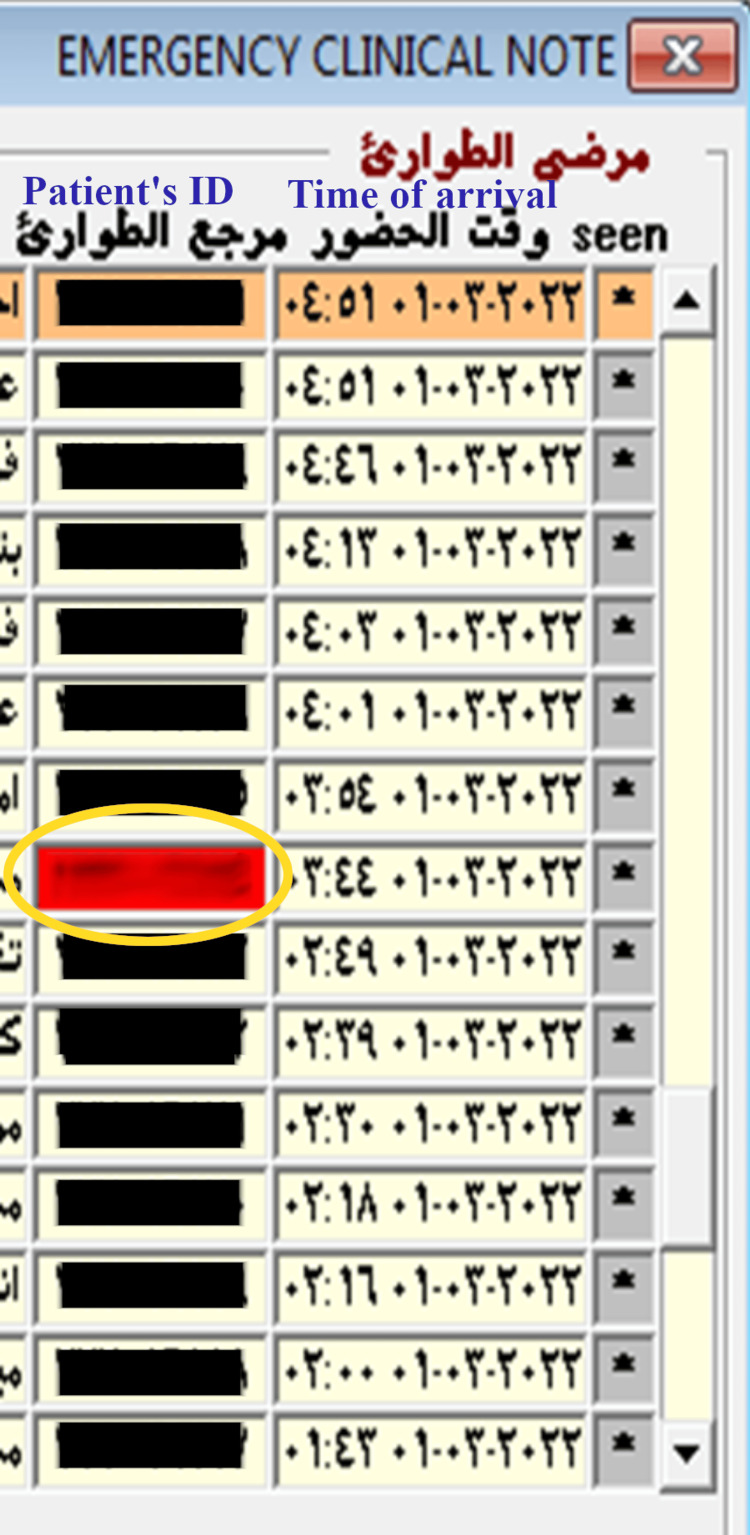
Red alert indicating the four-hour target breach.

**Figure 6:**
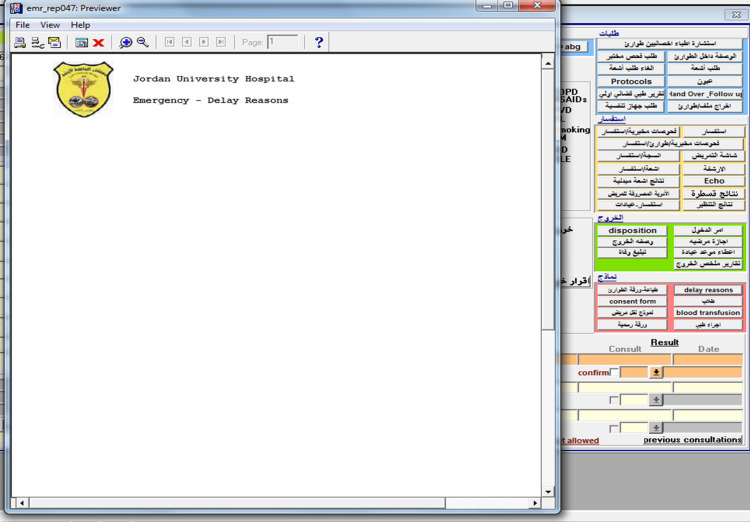
Delay reason input prompt.

**Figure 7:**
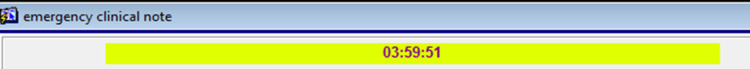
Patient’s four-hour timer.

**Figure 8:**
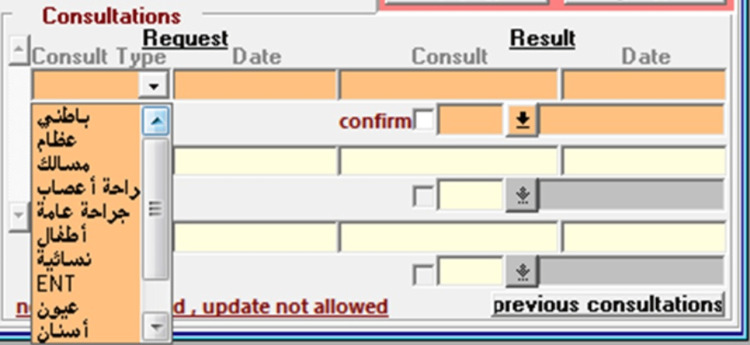
Consultations request window.

**Figure 9:**
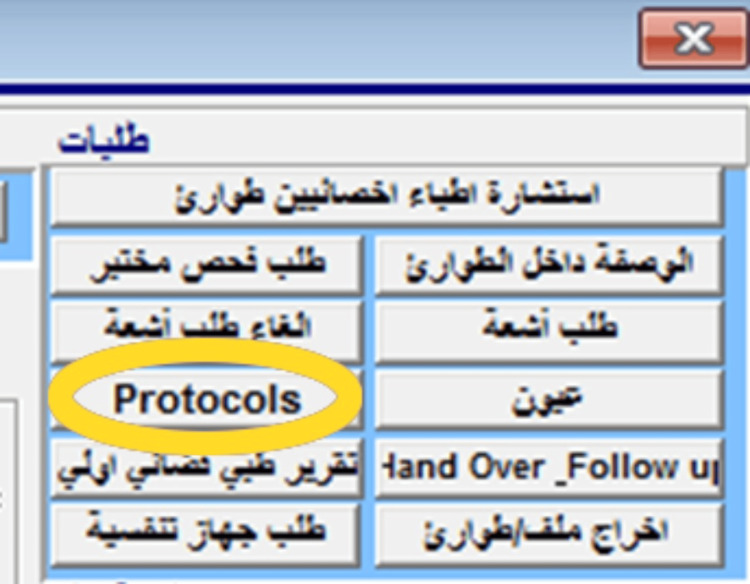
Protocol initiation button.

**Figure 10:**
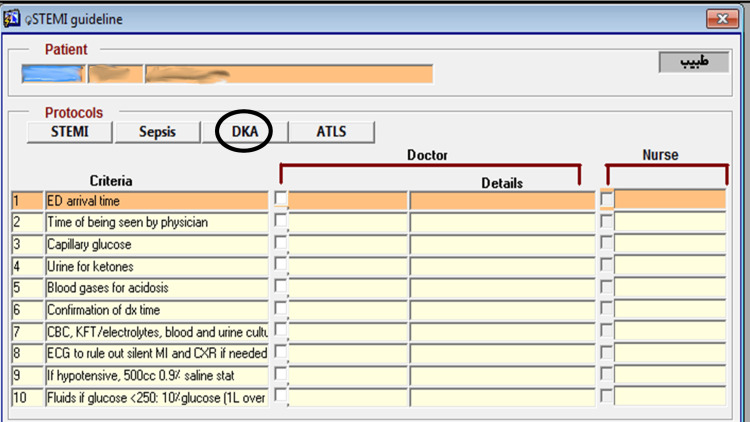
Diabetic ketoacidosis sample protocol.
